# Deep Vascular Imaging in Wounds by Two-Photon Fluorescence Microscopy

**DOI:** 10.1371/journal.pone.0067559

**Published:** 2013-07-02

**Authors:** Ciceron O. Yanez, Alma R. Morales, Xiling Yue, Takeo Urakami, Masanobu Komatsu, Tero A. H. Järvinen, Kevin D. Belfield

**Affiliations:** 1 Department of Chemistry, University of Central Florida, Orlando, Florida, United States of America; 2 The College of Optics and Photonics, University of Central Florida, Orlando, Florida, United States of America; 3 Sanford-Burnham Medical Research Institute at Lake Nona, Orlando, Florida, United States of America; 4 Department of Orthopaedic Surgery, Tampere City Hospital and Medical School, University of Tampere, Tampere, Finland; Medical College of Georgia, United States of America

## Abstract

Deep imaging within tissue (over 300 μm) at micrometer resolution has become possible with the advent of two-photon fluorescence microscopy (2PFM). The advantages of 2PFM have been used to interrogate endogenous and exogenous fluorophores in the skin. Herein, we employed the integrin (cell-adhesion proteins expressed by invading angiogenic blood vessels) targeting characteristics of a two-photon absorbing fluorescent probe to image new vasculature and fibroblasts up to ≈ 1600 μm within wound (neodermis)/granulation tissue in lesions made on the skin of mice. Reconstruction revealed three dimensional (3D) architecture of the vascular plexus forming at the regenerating wound tissue and the presence of a fibroblast bed surrounding the capillaries. Biologically crucial events, such as angiogenesis for wound healing, may be illustrated and analyzed in 3D on the whole organ level, providing novel tools for biomedical applications.

## Introduction

Microscopy of biological specimens deep within the tissue was limited to several hundred microns for several decades because visible light is severely scattered in biological tissue, leaving to histology the analysis of many relevant physiological events that occur deep within tissues of living organisms. Imaging within tissue (over 300 μm) at micrometer resolution has been achieved by two-photon fluorescence microscopy (2PFM).[1], [2] This technique has been useful in exciting endogenous and exogenous fluorophores in the skin.[3], [4], [5] A good example of the penetration capabilities of 2PFM was recently published, where an impressive 1.6 mm penetration depth was reported by 2PFM, in the cortex of a mouse brain.[6].

**Figure 1:**
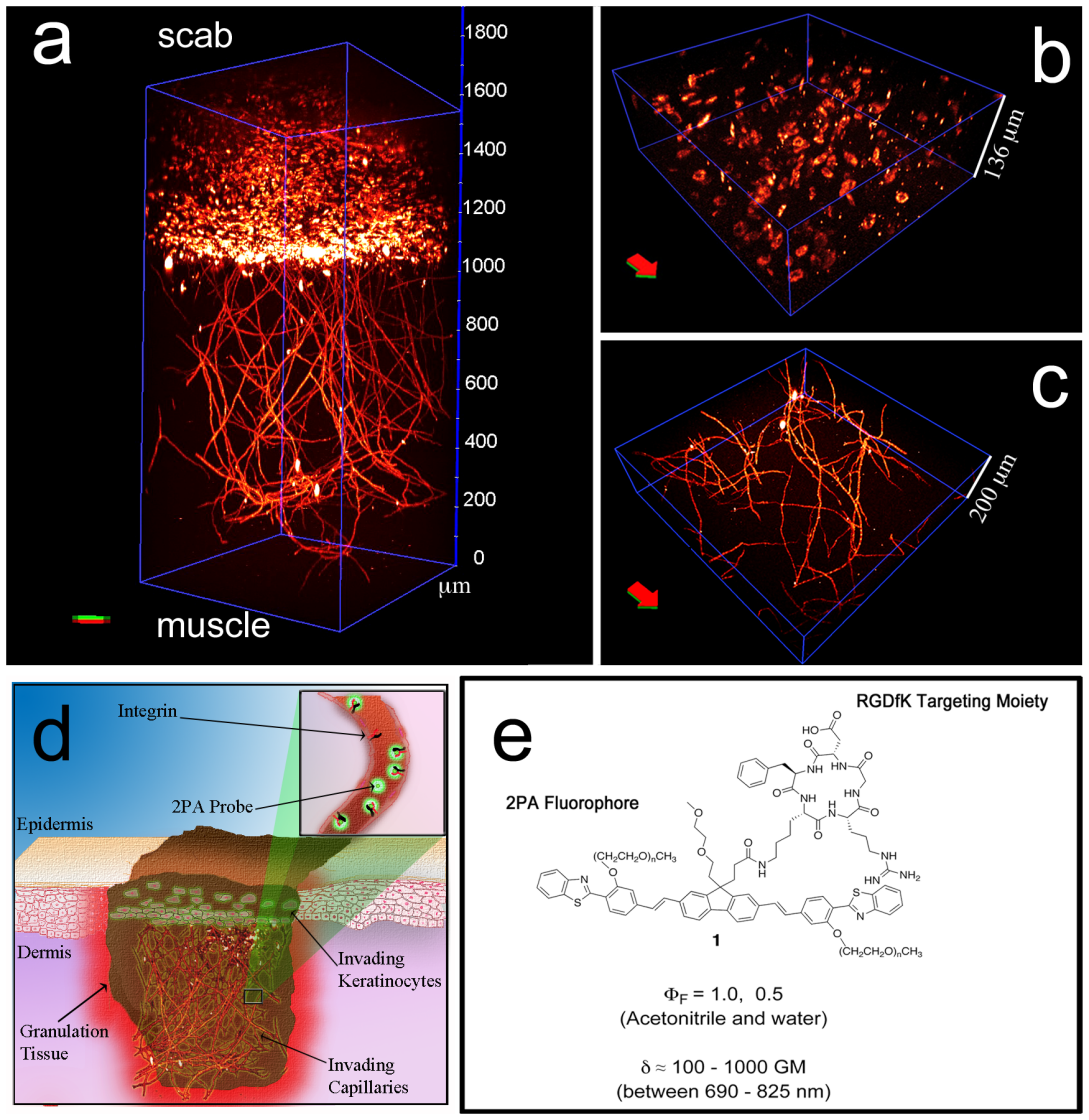
Two-photon fluorescence microscopy of the “whole-mounted” wounds was performed 800–825 nm to excite probe 1. 3-D reconstruction of invading capillaries from successive 1 μm optical sections. Vascular plexus extends from 0 to approximately 1100 μm and integrin-expressing cells from 1100 to 1600 μm (**a**). Integrin-expressing cells in optical section (1200–1336 μm). Probe **1** colocalized positively with some granulocytes that were costained with a Gr-1 antibody. Fibroblasts were stained with anti-fibroblast activation protein (FAP) antibody. Many fibroblasts within the fibrin clot colocalized positively with RGD probe-positive cells. Thus, the RGDfK moiety on probe **1** targeted endothelial cells and endothelial cell precursors. (**b**); capillaries in optical section (200–400 μm, **c**). Excitation (825 nm, 70 fs, 80 MHz, ≈ 5–7 mW) and collection was done perpendicularly to red-green arrow (excitation: from red to green, collection: from green to red). Objective: Leica 20×, 1.0 N. A., water. Depiction of a wound during the granulation tissue formation (**d**). During the proliferative phase fibroblast invade the fibrin clot, generating the granulation tissue. Angiogenesis accompanies the invading fibroblasts. Integrins are overregulated in fibroblasts (α_5_β_1_, α_v_β_6_) and in endothelial cells of capillaries (α_v_β_3,_ inset), which allows for the RGD-containing probe to interact with these cells. 2PA RGD-probe to target integrins (**e**).

The advantages of 2PFM have been exploited in imaging the skin and used in conjunction with one-photon reflection microscopy.[7] The skin, besides being the largest organ in the body, is a protective barrier that keeps other organs from being exposed to external harmful agents. As soon as an injury takes place on the skin, a clot is formed (fibrin clot) that acts as a temporary plug to seal it quickly. Within several hours following the insult, inflammatory cells invade the clot to fight against infection and to phagocytose necrotic cell debris. Several days later, the invasion of inflammatory cells is followed by capillaries and fibroblasts. Throughout the invasive neoangiogenesis that takes place during the wound healing process, endothelial cells up-regulate integrin α_v_β_3_, a specific adhesion receptor for migrating cells on their cell surface, but this integrin disappears from the blood vessels/wound once the revascularization is completed.^7–9^ Furthermore, during the early stages of wound healing, residing cells of the dermis that are in the immediate vicinity of the wound edges, which are otherwise relatively sedentary, become activated and invasive to form a matrix for what will become the repaired tissue (Fig. 1).

Many integrins, such as α_v_β_1,_ α_v_β_3_ and α_5_β_1_, are recognized by the Arg-Gly-Asp (RGD) motif found in many extracellular matrix proteins, i.e., expressed by their natural ligands.[8] Due to the restricted expression of α_v_β_3_ integrins on the angiogenic vasculature, linear and cyclic RGD-peptides specific for α_v_β_3_ integrins have been used for various purposes such as targeting drugs specifically to tumor vasculature.[9], [10] In this paper a custom-made 2PA-absorbing fluorescent probe was used to image invading, angiogenic capillaries within the wound. The integrin (cell-adhesion proteins expressed by invading cells) targeting characteristics of this fluorescent probe revealed new vasculature and fibroblasts up to ≈ 1600 μm within wound (neodermis)/granulation tissue in lesions made on the skin of mice. Reconstruction exposed the three dimensional (3D) architecture of the vascular plexus and the presence of a fibroblast bed surrounding the capillaries.

## Materials and Methods

Synthesis, purification, along with structural and photophysical characterization of probe **1** was performed as described previously.[11].

### Ethics Statement

All animal experiments were reviewed and approved by the institutional animal care and use committees of Department of Orthopedic Surgery, Medical School, University of Tampere, Finland.

### Wound Healing Model and Administration of Probe 1

Eight-week-old male BALB/c mice (weighing 23–25 g) were anesthetized with 4% isoflurane and 1.5 L/min of oxygen, and the anesthesia was maintained at ≈1.5% isoflurane at 1 L/min of oxygen. Skin was shaved, cleaned, and disinfected with betadine and 70% alcohol. Treatment trials were conducted on mice that had four circular, 6-mm diameter, full thickness (including *panniculus carnosus* muscle) excision wounds in the dorsal skin. The wounds were first marked by a biopsy punch and then cut with scissors. All skin wounds were left uncovered without a dressing. There are several reasons for using 4 wounds in each animal; one can assess the reproducibility of the results by analyzing four wounds. The policy of using 4 wounds instead of one also guarantees that only a limited number of animals was needed to perform the research. The wounds were oriented by placing two wounds on each side of the spine. Furthermore, four wounds increase the angiogenic response in the area making the model more representative to study angiogenesis-related issues.

**Figure 2:**
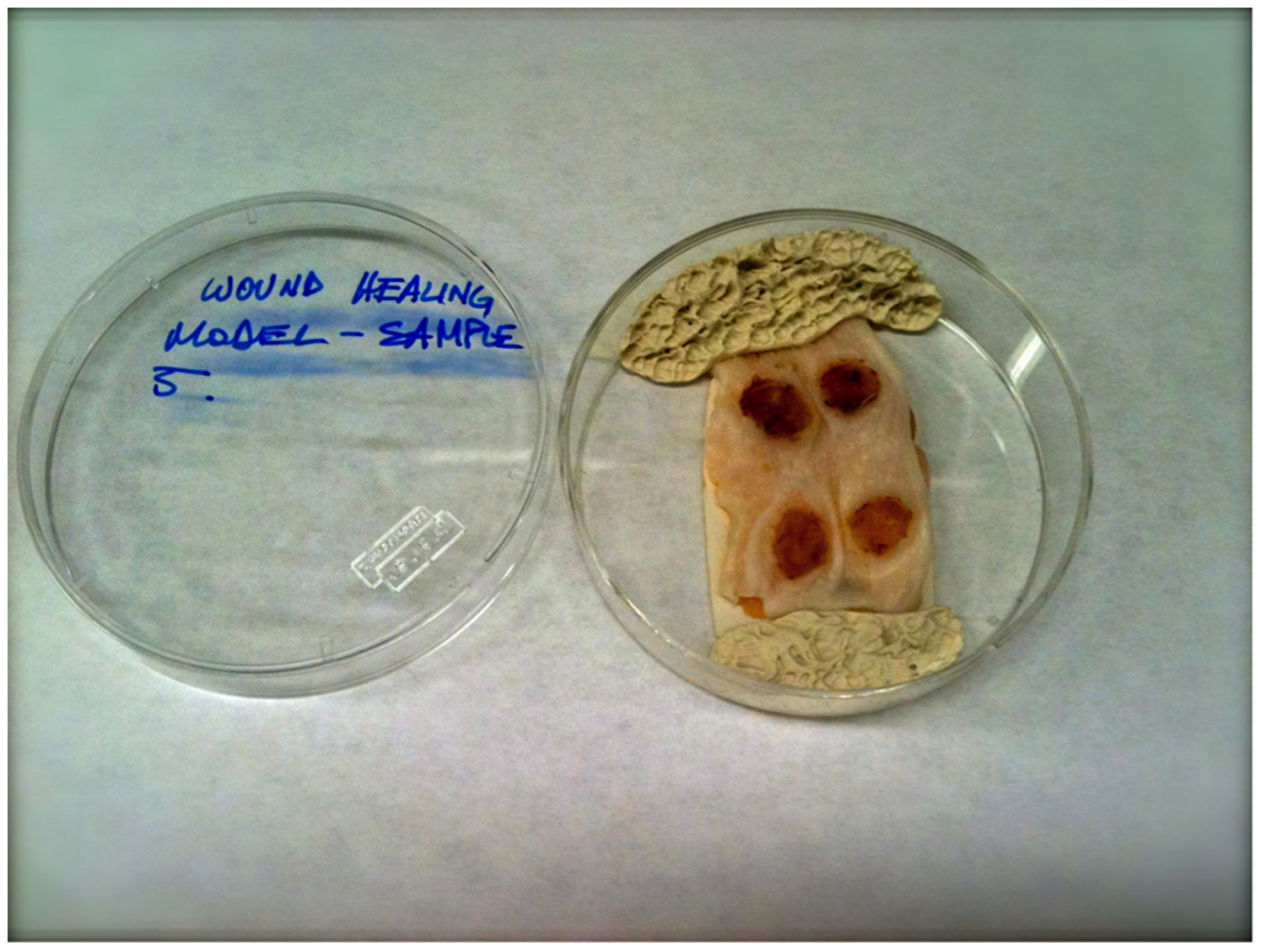
Excised wound healing sample (“whole-mounted”) as imaged and analyzed by 2PFM once it was excised from the mouse. The image shows four 7-day-old excision wounds (6 mm) with the scab tissue on top of the wounds in a petri capsule prior to imaging.

After 7 days post-wounding, the mice were injected with 200 μL of a 600 μM solution of probe **1** in PBS, and this was allowed to circulate for 2 h, then perfused first with 1×PBS +1% BSA and then with 4% paraformaldehyde (PFA) for fixation. Day 7 was chosen as the analysis point because the angiogenic response peaks at day 7 in wounds, i.e., there are more blood vessels at that point than at any other point during the healing process. Excision of a rectangular section of skin containing all wounds as well as underlying skeletal muscle was performed to ensure the uninterrupted wound architecture (Fig. 2). The “whole-mounted” sections were immobilized on filter paper, immersed in 4% PFA for additional O/N fixation, washed with physiological saline, and imaged from the internal and external faces of the wound.

### Micrososcopy

Two-photon fluorescence microscopy with probe **1** was performed on a Leica SP5 II equipped with a Coherent Chameleon Vision S laser source (prechirped compensated, 70 fs, 80 MHz). Micrographs of the whole-mounted fixed tissues were taken under the following conditions: excitation at 825 nm, emission external non-descanned PMT detectors (NDD). A 665 nm shortpass filter was incorporated into the scanhead of the microscope, to avoid excitation laser bleedthrough, and a bandpass barrier filter 457/50 was placed before the NDD. A 20×, 1.0 N. A. water immersion objective was used for *ex vivo* imaging. Segmentation analysis and 3D rendering was performed with Amira 5.4.1.

The tissue was excised in a fashion that a layer of paraspinal muscle beneath the skin is part of the excised block. This guarantees that the granulation tissue is not excised from the middle or ruptured during the tissue removal. Panniculus carnosus has nothing to do with the excision because panniculus carnosus is just a thin muscle layer in the middle of dermis. Excitation from the external face of the wound resulted in poor penetration (not shown) due to absorption by the scabs formed at the surface of the lesion, and the presence of fur also interfered with imaging the neovasculature. From the internal side, it was necessary to overcome collagen/elastin autofluorescence; yet the images were much clearer overall (Fig. 1, A–C). Imaging with shorter wavelengths (690–720 nm), where the δ_2PA_ ≈ 1000 GM, was also explored but the contrast was significantly diminished compared with the 800–825 nm excitation range. The loss of contrast was primarily due to the excitation of collagen and/or elastin autofluorescence in muscle fibers. Even though cross sections at 825 nm were roughly an order of magnitude lower than at 690 nm, penetration and contrast were significantly enhanced at this wavelength.

Segmentation analysis involved establishing a threshold for the pixels to be counted in each optical section; in all cases the pixels with lowest 5% and the highest 5% counts per second were discarded for the final volume tally. The volume, conformed by fluorescent pixels, was determined and divided by the total scanned volume to determine the vascular density.

### Immunohistochemistry

Formalin-fixed, paraffin-embedded tissue sections (5 µm thickness) were prepared from the skin wound model after whole mount two-photon excitation fluorescence imaging. Briefly, the skin was cut longitudinally at the middle of the wounds, mounted and paraffin embedded. Tissue sections were deparaffinized and antigen retrieved by Diva Decloacker (Biocare Medical, Concord, CA) at 120 °C for 4 min, blocked for unspecific binding by a species-matched 10% serum, and then stained with primary antibodies. The following primary antibodies were used: anti-CD34 (rat monoclonal IgG, clone MEC14.1∶100, BioLegend, San Diego, CA), anti-alpha smooth muscle actin (rabbit polyclonal IgG, ab5694, 1∶500) and Fibroblast activation protein, alpha (rabbit polyclonal IgG, ab53066, 1∶200, both from Abcam Cambridge, MA), anti-Mouse Mac-3 (rat monoclonal IgG, clone M3/84, 1∶100, BD Pharmingen, San Jose, CA). Alkaline phosphatase-conjugated with streptavidin (Vector Laboratories, Burlingame, CA) were used to detect primary antibodies in the combination with biotin-labeled species-specific secondary antibody anti-rat IgG (A10517, Invitrogen, Grand Island, NY) or anti-rabbit IgG (BA-1000, Vector Laboratories, Burlingame, CA) and visualized by fluorescent dye with VECTOR Red Alkaline Phosphatase Substrate Kit (SK-5100, Vector Laboratories). The localization of alkaline phosphatase substrate was observed by single-photon excitation fluorescence. Sections without primary antibodies and all other staining steps and chemicals were used as negative controls.

## Discussion

Understanding the complex dynamics of the wound healing process has traditionally relied on optical microscopy techniques of cells and tissue. Optical microscopy has been instrumental in understanding the process of wound healing, primarily via the staining (H&E) of tissue sections and fluorescence confocal microscopy analysis.[12], [13], [14] The limitation of penetration depths in conventional (one-photon absorption, 1PA) fluorescence microscopy has made tissue sectioning mandatory in the analyses of the wound healing process. However, both the fibrin clot and the early granulation tissue have “jelly-like” consistencies and are easily ruptured during the processing of the tissue, leading to the disrupted tissue architecture while the dense “scab” tissue (dead tissue) formed on top of the wounded area (on the top of the immature early granulation) makes it impossible to visualize the actual healing process from the top of the skin. Furthermore, the scab tissue also compromises the quality of the histological sections; vast areas of the wound are lost during the histological processing of the skin wounds.

The wound architecture was maintained virtually intact by keeping the surrounding tissue (primarily muscle) to support the sample during collection. The samples were “whole-mounted” (mounted without further sectioning) and analyzed by 2PFM once it was excised from the mouse (Fig. 2). The autofluorescence emanating from connective tissue of the muscle did not affect the analyses due to the high efficiency of the probe (high two-photon action cross section, δ.Φ_F_), which provided a high enough signal-to-noise ratio to reveal individual integrin-expressing fibroblasts and endothelial cells beyond 1000 μm.

Just as for linear absorption, the efficiency of two-photon absorption (2PA) can vary significantly from molecule to molecule. In order to take complete advantage of the virtues of 2PFM, fluorophores should have a very particular set of values that include high 2PA cross section, high fluorescence quantum yield, and low photo-decomposition quantum yields.[15] We recently reported the development of an efficient 2PA RGD-containing fluorescent probe (**1**) that has proven to be useful in imaging integrin sites in cells and tumor vasculature.[11] The probe consisted of two units: 1) a two-photon absorbing component that was designed to exhibit a high 2PA cross section and high fluorescence quantum yield; and 2) a cyclic-RGD peptide that targeted the probe toward α_v_β_3_ integrin expressed on the sprouting capillaries within the wound. The core of the 2PA chromophore was a fluorene molecule flanked by two benzothiazolyl styryl groups in positions 2- and 7-, constructing an A-π-π-π-A system, where A represents an electron-accepting moiety. An oligo-(ethylene glycol) (OEG) chain was incorporated to each styryl phenyl ring to improve the hydrophilicity of the probe. Cyclic RGDfK (c-RGDfK) peptide binds very specifically to α_v_β_3_ integrin.[16] Detailed linear and nonlinear photophysical characterization was previously reported for this probe.[11] The fluorescence quantum yields were 1.0, 1.0, and 0.5 when measured in chloroform, acetonitrile, and water, respectively. Although probe **1** is not symmetrical, its behavior was akin to that of compounds with C_2V_ symmetry, in the sense that the efficiency of its 2PA transition S_0_ →S_1_ is greatly reduced by the dipole selection rules.[17] Nonetheless, the 2PA cross section values were quite adequate for 2PFM, ranging from δ_2PA_ ≈ 100–1000 GM (between λ ≈ 690–825 nm). These values were crucial in obtaining the maximum possible contrast in the micrographs, particularly in this system where the fluorescence signal of the chromophore had to overcome the autofluorescence noise from the connective tissue in muscle that surrounded the fibrin clot.

**Figure 3:**
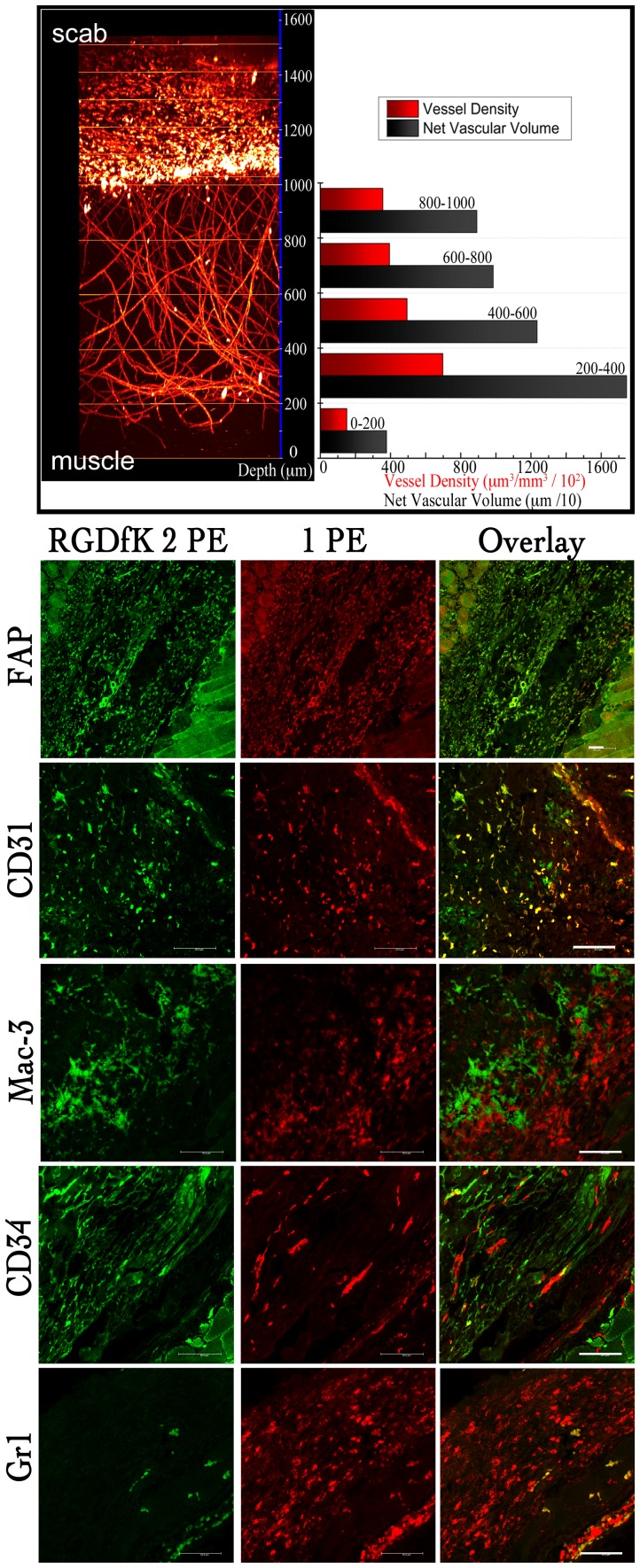
Segmentation analysis from 2PFM images of a wound healing specimen and immunohystostaining of tissue sections. Fluorescent pixels were used to account for the vasculature. Probe **1** enabled excellent contrast beyond 1 mm within tissue. Analysis was accomplished with Amira 5.4. Z-axis (blue), green lines delimit optical sections. Immunostaining of horizontal tissue sections of the wounds were used to screen for macrophages (Mac-3), integrin-expressing endothelial cells (CD31), endothelial cell precursors (CD34), granulocytes (Gr1), and fibroblasts (FAP). Fluorescence resulting from the two-photon excitation of probe **1** is shown in green while one-photon fluorescence is shown in red. Lower right is the muscle area and upper left is the area closest to the scab on all sections.

**Figure 4:**
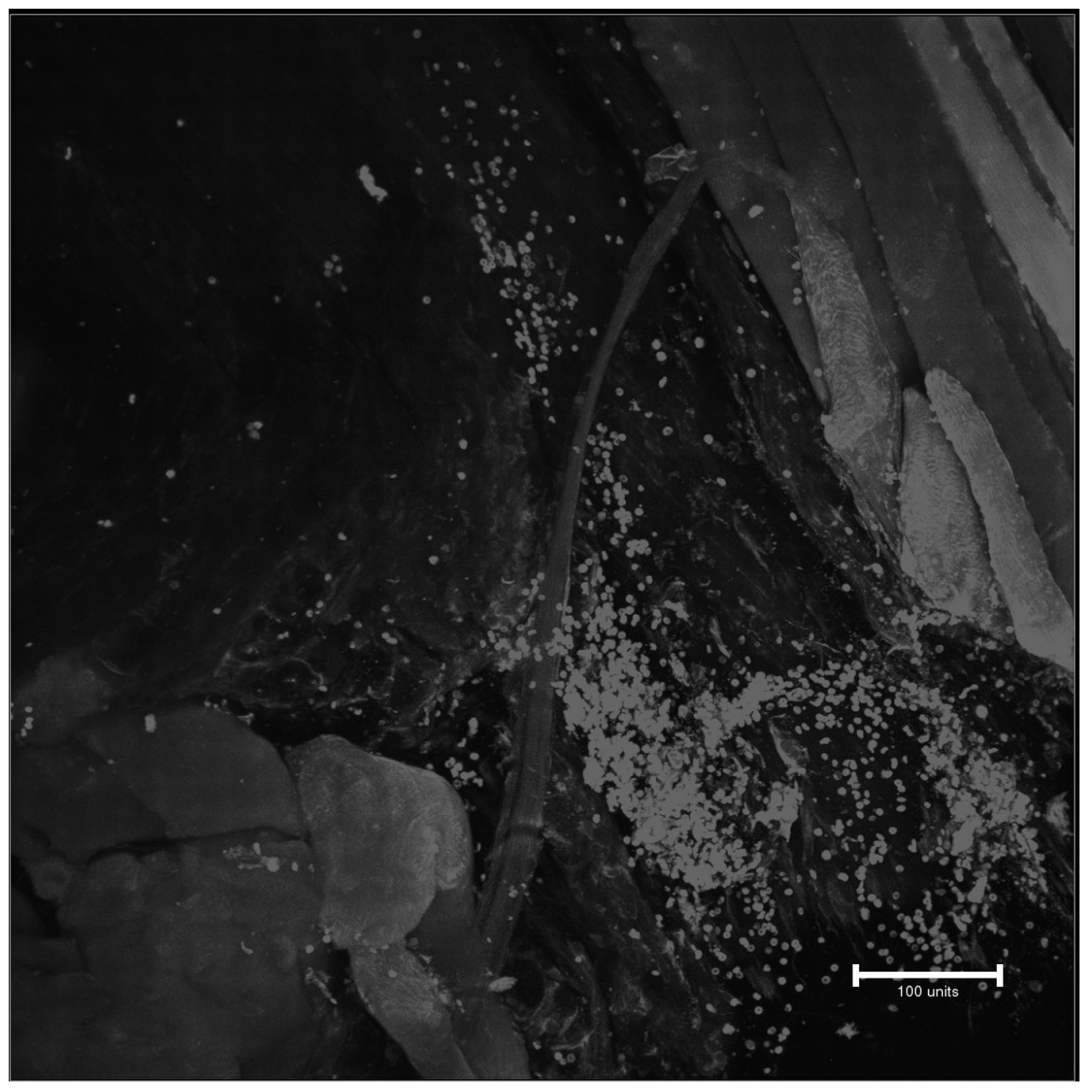
Two-photon fluorescence micrograph of the “whole-mounted” wounds without probe 1. Excitation at 825 nm, autofluorescence from connective tissue in muscle is observed.

Two of the main indicators of the proliferative phase in the granulation tissue during the wound healing process are the invasion of fibroblasts and the capillaries in fibrin clot.[12], [18] Within 2 to 3 days, when inflammation is receding, fibroblasts start to appear in the fibrin clot and are the predominating cells in the wound site after a week (Fig. 1). They rely on a fibrin/fibronectin scaffold to migrate into the wound. The main role of fibroblasts at this stage is to layout the collagen monomers. This early, loose granulation tissue, upon cross-linking of collagen, will form the firm collagen network at the later stages. This network is key for establishing the mechanical integrity for the disrupted tissue in later phases of the wound healing process. Angiogenesis is a concomitant event, or even precedes, the fibroblast invasion, providing both oxygen and nutrients the fibroblasts need in building the granulation tissue. Figure 3 (A, C, and D) illustrates new capillaries that have invaded the wound. Endothelial cells of new capillaries up-regulate α_v_β_3_ integrin, which is transiently expressed only at the tips of sprouting capillaries.[12] Thus, the peptide binding to α_v_β_3_ integrin is very specific for newly formed angiogenic blood vessels and can be used to illustrate the progress and extend of the angiogenesis as the tip of the sprouting capillaries are illuminated. Negative controls, shown in Figure 4, of wounds containing no dye did not show any structures.

After the whole-mount two-photon excitation fluorescence imaging of the excised wounds, immunofluorescence staining of horizontal tissue sections of the wounds were carried out to identify different populations of the wound bed; macrophages, integrin-expressing endothelial cells, granulocytes and fibroblasts in the granulation tissue (Fig. 3). The fluorescence resulting from two-photon excitation of probe **1** is shown in green and the one-photon fluorescence of each cell type marker is shown in red (Fig. 3). Macrophages were stained with a Mac-3 antibody conjugate; the Mac-3 antigen is upregulated by monocytes during their differentiation to macrophages. Colocalization of the Mac-3 conjugate with probe **1** was largely absent in this analysis. As expected, the entire fibrin clot was invaded by macrophages, whereas integrin positive cells had different cell distributions throughout the wound. A CD31 antibody conjugate was used to stain endothelial cells, partial co-localization with the signal from our probe confirmed that endothelial cells were successfully targeted. Furthermore, CD34 antibody, used to stain endothelial cells and endothelial cell precursors, showed partial colocalization with probe **1**. These results may suggest that not all capillaries were perfused and that probe **1** only labeled endothelial cells of perfused capillaries. This is, in turn, in agreement with the known fact that not all angiogenic blood vessels are perfused during angiogenesis.

Probe **1** colocalized positively with some granulocytes that were costained with Gr-1 antibody. Fibroblasts were stained with anti-fibroblast activation protein (FAP) antibody. FAP colocalization with probe **1** was positive. Many fibroblasts within the fibrin clot colocalized with RGD probe-positive cells, meaning that most of the probe **1** stained cells that were beyond 1100 micrometers in depth (from muscle) were fibroblasts. Thus, the RGDfK moiety on probe **1** targeted endothelial cells of invading capillaries (from 0–1100 micrometers), fibroblast, and endothelial cell precursors (from 1100–1600 micrometers). The probe then extravasated to the extracellular matrix to accumulate within the fibroblast-rich stroma. This observation is consistent with that fact that α_v_β_3_ integrin is expressed exclusively by the endothelial cells in the wound. Our results also suggest that the sprouting end of the new capillaries are quite leaky and that the probe binds to stromal cells that express other integrin receptors for RGD.

Reconstruction of the specimen in 3D (Fig. 1, A–C) showed the architecture of the intact capillary network and the relative position of surrounding stromal cells with respect to the invading capillaries. Our probe provides the possibility of carrying out analyses on the whole tissue-level in a 3D-format, i.e. the intact wound tissue architecture can be visualized by this method. Such imaging ability was previously unattainable with this level of detail in intact tissue. Furthermore, the probe effectively extravasated from the capillaries to the surrounding granulation tissue. The ability to extravasate and accumulate in the granulation tissue provides an unprecedented potential to identify tissue sequesters that are not perfused properly and could hamper tissue regeneration.

Segmentation analysis of the sample was performed throughout sections of the scanned volume to determine vascular densities (Fig. 3). Vascular densities varied significantly throughout the specimen, progressively becoming smaller as one approached the leading end of the capillaries where it reached its smallest value of ≈ 3500×µm^3^ of vessels per cubic millimeter of tissue.

In conclusion, probe **1** was useful in revealing RGD-positive, integrin-expressing cells and endothelial cells up to approximately 1600 μm deep within the specimen. *In silico* reconstruction showed high resolution 3D images of the intact structure of the vascular plexus in healing wounds. The RGD peptide-targeted 2PFM imaging overcame problems associated with histological preparation of fibrin clot for analyses of wounds. This technique offers the possibility of a novel method for cell tracking and monitoring of angiogenesis during the proliferative phase of wound healing, providing an attractive path forward towards *in vivo* wound healing studies as we were able to analyze the skin as a whole organ. Intravital 2PFM studies with custom-made imaging windows are currently being performed in our laboratory to image the wound healing process in real-time. This technology may not only be of substantial improvement for the reliable quantification and illustration of key biological processes taking place during the tissue regeneration in the skin, but also forge revolutionary opportunities to assess healing process in situations such as skin crafting and diabetes, where the re-vascularization of the craft/ischemic skin is the rate limiting step for regeneration to take place.

## References

[1] Denk W, Svoboda K (1997) Photon upmanship: Why multiphoton imaging is more than a gimmick. Neuron 18: 351–357. 9115730 10.1016/s0896-6273(00)81237-4

[2] Oheim M, Beaurepaire E, Chaigneau E, Mertz J, Charpak S (2001) Two-photon microscopy in brain tissue: Parameters influencing the imaging depth. J Neurosci Methods 111: 29–37. 11574117 10.1016/s0165-0270(01)00438-1

[3] Konig K, Riemann I (2003) High-resolution multiphoton tomography of human skin with subcellular spatial resolution and picosecond time resolution. J Biomed Opt 8: 432–439. 12880349 10.1117/1.1577349

[4] Schenke-Layland K, Riemann I, Damour O, Stock UA, Konig K (2006) Two-photon microscopes and in vivo multiphoton tomographs - powerful dicagnostic tools for tissue engineering and drug delivery. Adv Drug Del Rev 58: 878–896. 10.1016/j.addr.2006.07.004 17011064

[5] So PTC, Kim H, Kochevar IE (1998) Two-photon deep tissue ex vivo imaging of mouse dermal and subcutaneous structures. Opt Express 3: 339–350. 19384379 10.1364/oe.3.000339

[6] Kobat D, Horton NG, Xu C (2011) In vivo two-photon microscopy to 1.6-mm depth in mouse cortex. J Biomed Opt 16. 10.1117/1.3646209 22029361

[7] Chen W-L, Chou C-K, Lin M-G, Chen Y-F, Jee S-H, et al. Single-wavelength reflected confocal and multiphoton microscopy for tissue imaging. J Biomed Opt - 14: 054026. 10.1117/1.3247157 19895128

[8] Ruoslahti E (1996) Rgd and other recognition sequences for integrins. Annu Rev Cell Dev Biol 12: 697–715. 8970741 10.1146/annurev.cellbio.12.1.697

[9] Hirano Y, Kando Y, Hayashi T, Goto K, Nakajima A (1991) Synthesis and cell attachment activity of bioactive oligopeptides - rgd, rgds, rgdv, and rgdt. J Biomed Mater Res 25: 1523–1534. 1724445 10.1002/jbm.820251209

[10] Dechantsreiter MA, Planker E, Matha B, Lohof E, Holzemann G, et al (1999) N-methylated cyclic rgd peptides as highly active and selective alpha(v)beta(3) integrin antagonists. J Med Chem 42: 3033–3040. 10447947 10.1021/jm970832g

[11] Morales AR, Luchita G, Yanez CO, Bondar MV, Przhonska OV, et al (2010) Linear and nonlinear photophysics and bioimaging of an integrin-targeting water-soluble fluorenyl probe. Org Biomol Chem 8: 2600–2608. 20376401 10.1039/b925934a

[12] Brooks PC, Clark RAF, Cheresh DA (1994) Requirement of vascular integrin alpha(v)beta(3) for angiogenesis. Science 264: 569–571. 7512751 10.1126/science.7512751

[13] Cavani A, Zambruno G, Marconi A, Manca V, Marchetti M, et al (1993) Distinctive integrin expression in the newly forming epidermis during wound-healing in humans. J Invest Dermatol 101: 600–604. 8409530 10.1111/1523-1747.ep12366057

[14] Jarvinen TAH, Ruoslahti E (2010) Target-seeking antifibrotic compound enhances wound healing and suppresses scar formation in mice. Proc Natl Acad Sci U S A 107: 21671–21676. 21106754 10.1073/pnas.1016233107 PMC3003105

[15] Pawlicki M, Collins HA, Denning RG, Anderson HL (2009) Two-photon absorption and the design of two-photon dyes. Angew Chem Int Ed 48: 3244–3266. 10.1002/anie.200805257 19370705

[16] Pfaff M, Tangemann K, Muller B, Gurrath M, Muller G, et al (1994) Selective recognition of cyclic rgd peptides of nmr defined conformation by alpha-ii-beta-3, alpha-v-beta-3, and alpha-5-beta-1 integrins. J Biol Chem 269: 20233–20238. 8051114

[17] Peticola.Wl (1967) Multiphoton spectroscopy. Annu Rev Phys Chem 18: 233-&.

[18] Stadelmann WK, Digenis AG, Tobin GR (1998) Physiology and healing dynamics of chronic cutaneous wounds. Am J Surg 176: 26S–38S. 9777970 10.1016/s0002-9610(98)00183-4

