# Correction: Deep Vascular Imaging in Wounds by Two-Photon Fluorescence Microscopy

**DOI:** 10.1371/annotation/59bcbe81-eddd-46a4-90dc-88c1ea70df72

**Published:** 2014-01-22

**Authors:** Ciceron O. Yanez, Alma R. Morales, Xiling Yue, Takeo Urakami, Masanobu Komatsu, Tero A. H. Järvinen, Kevin D. Belfield

The institutional affiliation number 4 for the sixth author, Tero A. H. Järvinen, is incorrect. The correct affiliation is: Departments of Orthopedics & Traumatology and Anatomy, Medical School, University of Tampere and Tampere University Hospital, Finland.

In addition, funding organizations for the sixth author were incorrectly omitted from the Funding Statement. The following sentence should be included with the Funding Statement: "T.A.H.J. is funded by the Academy of Finland, Instrumentarium Research Foundation, Sigrid Juselius Foundation and Finnish Medical Foundation."

